# Melanocortin 1 Receptor-Signaling Deficiency Results in an Articular Cartilage Phenotype and Accelerates Pathogenesis of Surgically Induced Murine Osteoarthritis

**DOI:** 10.1371/journal.pone.0105858

**Published:** 2014-09-05

**Authors:** Julia Lorenz, Elisabeth Seebach, Gerit Hackmayer, Carina Greth, Richard J. Bauer, Kerstin Kleinschmidt, Dominik Bettenworth, Markus Böhm, Joachim Grifka, Susanne Grässel

**Affiliations:** 1 Experimental Orthopedics, University Hospital of Regensburg, Regensburg, Bavaria, Germany; 2 Research Centre for Experimental Orthopedics, Orthopedic University Hospital Heidelberg, Heidelberg, Baden-Württemberg, Germany; 3 Oral and Maxillofacial Surgery, University Hospital of Regensburg, Regensburg, Bavaria, Germany; 4 TIP Immunology, Merck Serono Global Research & Development, Darmstadt, Hessen, Germany; 5 Medical Hospital B, University Hospital of Münster, Münster, North Rhine-Westphalia, Germany; 6 Dermatology, University Hospital of Münster, Münster, North Rhine-Westphalia, Germany; 7 Orthopedic Surgery, University Hospital of Regensburg, Bad Abbach, Bavaria, Germany; Université Jean Monnet, France

## Abstract

Proopiomelanocortin-derived peptides exert pleiotropic effects via binding to melanocortin receptors (MCR). MCR-subtypes have been detected in cartilage and bone and mediate an increasing number of effects in diathrodial joints. This study aims to determine the role of MC1-receptors (MC1) in joint physiology and pathogenesis of osteoarthritis (OA) using MC1-signaling deficient mice (Mc1re/e). OA was surgically induced in Mc1re/e and wild-type (WT) mice by transection of the medial meniscotibial ligament. Histomorphometry of Safranin O stained articular cartilage was performed with non-operated controls (11 weeks and 6 months) and 4/8 weeks past surgery. µCT–analysis for assessing epiphyseal bone architecture was performed as a longitudinal study at 4/8 weeks after OA-induction. Collagen II, ICAM-1 and MC1 expression was analysed by immunohistochemistry. Mc1re/e mice display less Safranin O and collagen II stained articular cartilage area compared to WT prior to OA-induction without signs of spontaneous cartilage surface erosion. This MC1-signaling deficiency related cartilage phenotype persisted in 6 month animals. At 4/8 weeks after OA-induction cartilage erosions were increased in Mc1re/e knees paralleled by weaker collagen II staining. Prior to OA-induction, Mc1re/e mice do not differ from WT with respect to bone parameters. During OA, Mc1re/e mice developed more osteophytes and had higher epiphyseal bone density and mass. Trabecular thickness was increased while concomitantly trabecular separation was decreased in Mc1re/e mice. Numbers of ICAM-positive chondrocytes were equal in non-operated 11 weeks Mc1re/e and WT whereas number of positive chondrocytes decreased during OA-progression. Unchallenged Mc1re/e mice display smaller articular cartilage covered area without OA-related surface erosions indicating that MC1-signaling is critical for proper cartilage matrix integrity and formation. When challenged with OA, Mc1re/e mice develop a more severe OA-pathology. Our data suggest that MC1-signaling protects against cartilage degradation and subchondral bone sclerosis in OA indicating a beneficial role of the POMC system in joint pathophysiology.

## Introduction

Osteoarthritis is an age-related and/or trauma-induced multi-factorial, slowly progressing degenerative disorder of the synovial joints culminating in the irreversible destruction of the articular cartilage. Clinical symptoms of OA appear in more than 10% of the world population affecting almost everyone over the age of 65. As a consequence of the increasing longevity and obesity in the European Community, the burden caused by OA rapidly grows substantially influencing life quality of the affected individuals with enormous costs to the health care system. Current therapeutic strategies seek to ameliorate pain and increase mobility, however, to date none of them halts disease progression or regenerates damaged cartilage. Thus, there is an ultimate need for the development of non-invasive treatments that could substitute joint replacement. OA exact aetiology is still unclear. Genetic disorders, limb mal-alignment and overuse as well as metabolic problems (obesity, immune responses, inflammation) play an important role in the onset of OA. [1]–[4]. Besides chondrocytes and cartilage as mediators of OA also other cells and tissues of the joint like synovium or subchondral bone modulate OA-pathogenesis. Subchondral bone and articular cartilage are separated through the tide mark region and have a close relationship during progression of OA. Abnormal calcification of this tide mark region during OA leads to a decrease in cartilage thickness and increased subchondral plate thickness [5] preceded by increased turnover of subchondral bone, thinning trabecular structures, sclerosis of the subchondral plate, bone marrow lesions and subchondral bone cysts [6], [7]. Alterations in subchondral bone remodeling add to biomechanical changes and enhance OA progression [8], [9].

Proopiomelanocortin (POMC) is a multifunctional precursor protein for several biologically active hormones which include the melanocyte-stimulating hormones (α-, β- and γ-MSH) and adrenocorticotropic hormone (ACTH). These peptides play an important role in a diversity of physiological processes including energy homeostasis, adrenal function, sexual activity, thermoregulation, nociception, exocrine gland activity, immune function, and pigmentation. Although originally characterized as neurohormones induced by stressful signals in context of the classical hypothalamic-pituitary-adrenal (HPA) stress axis, it is now established that POMC and its derived peptides can also be autonomously generated in a number of peripheral tissues, e. g. the skin and diathrodial joints. Here, receptors for POMC peptides, the melanocortin receptors (MCR) have been identified in various resident cell types where they elicit biological effects far beyond the initially identified action of their ligands. A very fascinating aspect of POMC-derived peptides is their immunomodulatory potential, especially that of melanocortin-derived peptides [10]–[12].

The melanocortin peptides ACTH as well as α-, β- and γ-MSH bind with high affinity to melanocortin receptors [13]. Five MCR subtypes, MC1 to MC5, have been cloned and which bind melanocortins with different affinities. With respect to diathrodial joints, initially the MC3 was detected in rat bone marrow stroma-like cells and in chondrocytes isolated from ribcages of young rats [14]. Our group recently demonstrated the presence of MC1, MC2 and MC5 transcripts in human articular chondrocytes derived from patients with OA [15]. Protein expression of the MC1 in these cells was confirmed on OA cartilage explants. Here, chondrocytes located in the middle and deep cartilage layers were immuno-reactive for MC1 while chondrocytes in the superficial zone were mostly negative. Treatment of these chondrocytes with α-MSH was associated with functional coupling as shown by cAMP assays but not with a Ca^2+^ response. The detection of MC1 in human articular chondrocytes is in accordance with the observation that also a human chondrosarcoma cell line, likewise expresses functional MC1 [16]. In addition, transcripts of all five MCR were found in normal human osteoblasts as well as in MG63 and SAOS-2 osteosarcoma cells, albeit not all receptors were present in each cell type [17].

For analyzing the role of the MC1 in pathogenesis of OA, we have induced OA surgically in mice using the model of destabilization of the medial meniscus (DMM) as it induces OA with great ease and reproducibility. It is less invasive than the anterior cruciate ligament transaction (ACLT) procedure and resembles more closely slowly progressive human OA. It has been shown to be sufficiently sensitive to detect significant protection against OA progression in ADAMTS-5 and IL-1β knockout joints at 4–8 weeks following DMM [18], [19]. We assume that the DMM-OA model will thus be sensitive enough to allow evaluation of the hypothesized chondroprotective role of endogenously expressed Mc1r in murine articular cartilage.

As a tool for analysis of the melanocortin system in OA, we have used yellow colored MC1 signaling-deficient (Mc1r*e/e*) mice. The Mc1r*e/e*- recessive yellow allele (e) results from a frameshift between the IV. and V. transmembrane domain that produces a prematurely terminated, non-functioning MC1 which does not functionally couple to adenylate cyclase [20] but is still expressed and transported to the cell membrane.

## Materials and Methods

### Mouse model

To evaluate the role of MC1 signaling during osteoarthritis pathogenesis, Mc1re/e mice on a C57BL/6 background were used [20]. C57BL/6 mice (Charles River) served as wild type (WT) control group.

### Study design

Right knees of Mc1re/e mice and WT received DMM while left knee served as sham control with just the surgical access set. Cartilage matrix was evaluated after histological assessment with scoring [21] and histomorphometry whereas changes in subchondral bone were evaluated with X-ray scoring and µCT. The experimental protocol was approved by the local animal experimental ethics committee (Az.: 54-2532.1-36/11) and all procedures were performed according to the European Laboratory Animal Science Guidelines.

For histological assessment, 22 male WT and 21 male Mc1re/e mice were included in study. 11 weeks (5 WT, 4 Mc1re/e) and 6 months (5 WT, 5 Mc1re/e) old, male mice received no DMM and represent non-operated control group whereas 12 WT and 12 Mc1re/e received DMM (6 WT and 6 Mc1re/e mice for 4 and 8 weeks each). To analyze OA progression, Safranin O/Fast green stained knee joint sections were scored and evaluated morphometrically.

For X-ray scoring and µCT analysis, 7 WT (2 female, 5 male) and 7 Mc1re/e (2 female, 5 male) mice received DMM. Changes in epiphyseal bone micro-architecture and osteophyte number were assessed directly before surgery (day 0) and 4 and 8 weeks after OA-induction.

### Surgically induced osteoarthritis

10–11 week old mice were anaesthetized by intraperitoneal injection of ketamine-hydrochloride (90–120 mg/kg body weight, Bela-pharma, Vechta) and Xylazin (6–8 mg/kg body weight, Serumwerk Bernburg). DMM was performed as described previously [19] from Dr. med. vet. Gerit Hackmayer (doctor of veterinary medicine). In both knees, a 3 mm longitudinal incision between the distal patella and proximal tibia plateau was set and the joint capsule was opened medial to the patellar tendon. In the right knee, the medial meniscotibial ligament (MMTL) was carefully transected, while in left knee MMTL was visualized but not transected (sham surgery). Joint capsule, subcutaneous layer and skin were closed and mice were allowed full activity. Mice were administered subcutaneous sodium chloride infusion (10 ml/kg body weight) and Buprenorphin (0.09 mg/kg body weight, Bayer vital) 12 and 24 h after operation in a longitudinal study.

### Histological preparation, OA-scoring and histomorphometry

For histological preparation and morphometry, we correspond mainly to the recommendation of OARSI [21]. Knee joints were fixed for 18–24 h in 4% paraformaldehyde and decalcified with 25% EDTA for 2 weeks at room temperature and embedded in paraffin. 5 µm frontal sections through the weight bearing area of each knee joint were taken. 5–6 sections in 60–80 µm intervals from 6 mice per time point were stained with Safranin O, Weigerts iron haematoxylin and fast green and scored by two independent observers.

Additionally, histomorphometry was performed with the same sections used for OA-scoring using graphic tablet Bamboo (Wacom) and image J software (NIH, Bethesda). On sections of knee joints from non-operated mice, total cartilage area was determined in pixel. On sections of knee joints from non-operated mice, 4 and 8 weeks after OA-induction, total articular cartilage area and cartilage area without cells/Safranin O staining (destroyed cartilage) was determined in pixel. Destroyed cartilage area was related to total cartilage area which was set as 100%.

For both methods medial regions of the knee joints were analyzed and mean values of femur and tibia scores were included in statistical analysis.

### Localization of MC1, collagen II, matrix metalloproteinase 13 (MMP-13), aggrecan neoepitope DIPEN(341)/(342)FFGVG (DIPEN) and intracellular adhesion molecule 1(ICAM-1)

For each antigen, 1–3 representative sections in the weight bearing area of 4–6 different mice per time point were chosen and included in staining. For immunostaining of MC1, collagen II, MMP-13, DIPEN and ICAM-1, following antibodies were used: rabbit anti-mouse mc1r [22], mouse anti-mouse collagen II (DSHB), rabbit-anti MMP-13 (Abcam), rabbit anti-DIPEN (a generous gift from Amanda Fosang (University of Melbourne)) and rabbit-anti-ICAM (BioVision).

Sections were deparaffinized, rehydrated and endogenous peroxidase activity was blocked with 3% H_2_O_2_ for 5 min at RT. For MC1, collagen II, MMP-13 and DIPEN staining, sections were pre-incubated with protease XXIV (0.05% in PBS for 10 min at 37°C, Sigma-Aldrich) and with testicular hyaluronidase (0.1% in acetate buffer pH 6.0 for 90 min at 37°C, Sigma-Aldrich). For staining of specimen with antibodies against ICAM, heat induced epitope retrieval was performed for 20 min at 98 °C in 10 mM sodium citrate buffer, pH 6.0. This was followed by a cooling period at room temperature for 20 min.

Sections were incubated over night at 4°C with MC1, MMP-13, DIPEN or ICAM-1 antibodies (1∶75 dilution in blocking solution for MC1-ab, 1∶600 for MMP-13-ab, 1∶5000 for DIPEN-ab and 1∶100 for ICAM-1-ab), after blocking with 1% bovine serum albumin (Biomol) and 5% swine serum (Dako). 1 h incubation with a biotinylated swine anti-rabbit (1∶500 diluted in PBS, Dako) was followed by an incubation with streptavidin-peroxidase and buffered substrate solution containing H_2_0_2_ and 3,3-diaminobenzidine chromogen solution (Dako). The secondary antibody for the ICAM-1-staining was provided with the Envision kit (Dako) according to manufacturer's instruction (incubation time was 30 min). Sections were counterstained with hematoxylin modified after Gil (Merck), dehydrated and mounted.

For staining of sections with antibodies against collagen II, a commercial biotinylation kit was used according to manufactor's instructions (Dako) with a 1∶125 dilution of primary antibody. Sections were counterstained with Weigert's hematoxylin, dehydrated and mounted.

For MC1, collagen II, MMP-13, DIPEN and ICAM-1 staining, images from both groups of each condition were compared and evaluated microscopically. To quantify collagen II, MMP-13, DIPEN and ICAM-1 stained sections, a scoring system was applied. For collagen II stained sections, percentage of stained cartilage area (see Tab. 1), for MMP-13 staining, percentage of positive non-hypertrophic articular chondrocytes (see Tab. 2) and for ICAM-1 and DIPEN, percentage of stained articular chondrocytes was estimated (see Tab. 3). To evaluate scoring systems for collagen II, MMP-13, DIPEN and ICAM-1 staining, intra- and inter-observer agreement was determined with cohens kappa coefficients. Therefore 7 independent observers scored 10 frontal sections of each staining and 4 of them scored the same images after 1 week again. To score the images, observers got no further information except for scoring tables (Tab. 1–3) and 4 exemplary images with scores according to [21] (Fig. S4).

**Table 1:** Scoring system for collagen type II immunohistochemistry.

Grade	collagen type II staining in articular cartilage
**0**	articular cartilage is not stained
**1**	<25% of articular cartilage area is stained
**2**	25–50% of articular cartilage area is stained
**3**	50–75% of articular cartilage area is stained
**4**	>75% of articular cartilage area is stained

**Table 2:** Scoring system for MMP-13 immunohistochemistry.

Grade	MMP-13 stained non-hypertrophic chondrocytes in articular cartilage
**0**	no chondrocytes are stained
**1**	<25% of chondrocytes are stained
**2**	25–50% of chondrocytes are stained
**3**	50–75% of chondrocytes are stained
**4**	>75% of chondrocytes are stained

**Table 3:** Scoring system for ICAM-1 and DIPEN immunohistochemistry.

Grade	ICAM-1/DIPEN stained chondrocytes in articular cartilage
**0**	no chondrocytes are stained
**1**	<25% of chondrocytes are stained
**2**	25–50% of chondrocytes are stained
**3**	50–75% of chondrocytes are stained
**4**	>75% of chondrocytes are stained

Sections incubated with appropriate isotype control antibody served as negative control and showed no staining (Fig. S1).

### X-ray Scoring

Mice were scanned under anesthesia by intraperitoneal injection of ketamine hydrochloride (Ketavet: 120 mg/kg body weight, Pfizer, Berlin, Germany) und medetomidine hydrochloride (Domitor: 0.5 mg/kg body weight, Orion Pharma, Hamburg, Germany) in the Sky-Scan 1076 in vivo x-ray microtomograph (Skyscan, Antwerpen). Anesthesia was antagonized after µCT-scan by subcutaneous administration of atipamezole hydrochloride (Antisedan: 2.5 mg/kg body weight, Orion Pharma, Hamburg, Germany). 2 Mc1re/e mice died due to anesthesia reducing the group size to 5 mice (1 female, 4 male). Legs were hold in a stretched position by a small styrofoam cylinder taped between hind legs. Following settings were used for µCT analysis: 1 mm aluminium filter, voxel size 8.85 µm, voltage 40 kV, current 250 µA, exposure time 2550 ms, frame averaging 2 [23]. Data were recorded every 1.5 degree rotation step through 180 degrees. Both legs were scanned in the same scan session and a shutter was set to reduce X-ray exposure time (<20 min).

### Micro-CT analysis

Reconstruction of X-ray scans was performed using NRecon software (version 1.6.3.2, Skyscan). Datasets were separated using DataViewer (Skyscan). Knees were manually analyzed for osteophytes based on the reconstructed images in transaxial, sagittal and coronal plane. Osteophytes were defined as abnormal bony projections along the bony margin of the knee joints appearing after 4 and 8 weeks of OA progression. Presence of osteophytes was assessed by two independent investigators. For further 3D analysis, tibia plateau was oriented along the growth plate and tibial diaphysis according to transaxial, sagittal and cortical plane. A volume of interest (VOI) of 400×400×100 slices per knee was set. Bone parameters were analyzed with CTAn analysis software (Skyscan) with a threshold set at 55 to define mineralized callous tissue. An interpolated VOI was set along the outer bone boundaries of the tibia plateau excluding the growth plate. For evaluating bone density the mean grayscale within the VOI was determined. Changes in bone nature were calculated regarding following 3D parameters: bone volume/tissue volume (BV/TV), trabecular number (Tb.N), trabecular thickness (Tb.Th) and trabecular separation (Tb.Sp). Parameters are shown following ASBMR nomenclature [24].

### Statistical analysis

For statistical evaluation of histological and morphometric scoring non-parametric Mann-Whitney-U test was used. A two-tailed significance value of p<0.05 was considered statistically significant. Data analysis was performed with GraphPad Prism 5 (San Diego).

After consultation of a statistician, the intra- and inter-observer agreement of histological scoring systems was calculated using Cohens kappa coefficient. Hereby, a kappa value of 0 corresponds to no agreement and a kappa value of 1.0 to complete agreement. Kappa values between 0–0.20 indicate slight agreement, 0.21–0.40 indicate fair agreement, 0.41–0.60 indicate moderate agreement, 0.61–0.80 indicate substantial agreement and 0.81–1.00 indicate excellent agreement. [25]. Data analysis was performed with SPSS for Windows 22.0 (SPSS Inc., Chicago).

For µCT analysis all parameters were referred to the respective value at day 0. For statistical comparison of Mc1re/e mice (n = 5) with WT mice (n = 7), Mann-Whitney-U Signed-Rank tests was conducted. For comparison of time points (day 0, 4 weeks and 8 weeks) Friedman-overall-test with post-hoc Wilcoxon-tests was conducted. Only if Friedman-overall-test indicated significance, data were analyzed post-hoc. Comparison of the right and the left knee was done by a paired Wilcoxon-test. A two-tailed significance value of p<0.05 was considered statistically significant. Data analysis was performed with SPSS for Windows 16.0 (SPSS Inc., Chicago).

## Results

### Morphometric analysis of knee joint articular cartilage from non-operated Mc1re/e and WT mice

**Figure 1:**
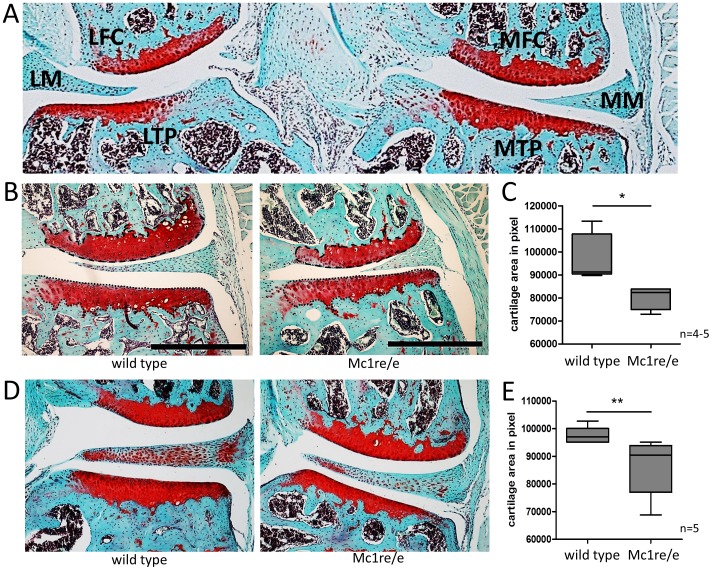
Histomorphometric comparison of knee joint morphology between non-operated Mc1re/e and WT mice. A) Overview of Safranin O/Fast green stained frontal section of a right mouse knee joint from a non-operated 11 week old Mc1re/e mouse (40× magnification). Lateral femoral condyle (LFC), lateral tibial plateau (LTP) and lateral meniscus (LM) as well as medial femoral condyle (MFC), medial tibial plateau (MTP) and medial meniscus (MM) are labeled. For osteoarthritis induction with DMM, the medial meniscotibial ligament is transected. B–E) Histological evaluation of cartilage area was performed with Safranin O/Fast green stained frontal sections of right knee joints from non-operated 11 weeks old and 6 months old WT and Mc1re/e mice. B+D) Medial, tibial (dotted line) and femoral (broken line) Safranin O stained cartilage was circuited with Bamboo tablet. C+E) Cartilage area of Mc1re/e mice was significantly smaller in 11 weeks (p = 0.0159) and 6 months old (p = 0.0079) animals compared to cartilage area of WT mice. For statistical analyses mean values of tibial and femoral cartilage areas of 5–6 section per knee joint was determined. Data are presented as boxplots reflecting the 25th and 75th percentile as boxes, the median as horizontal line and minimum and maximum values as whiskers. Bars = 500 µm, * p<0.05 and ** p<0.01 wild type vs. Mc1re/e.

Fig. 1A depicts representative Safranin O/fast green stained image from a frontal section through a mouse knee joint. For OA – induction always the meniscotibial ligament of the medial meniscus (MM) was transected. For further evaluation always the medial femoral condyle (MFC) and the medial tibial plateau (MTP) were used. Articular cartilage morphology was assessed by morphometric evaluation of Safranin O stained area of knee joints of non-operated 11 weeks (Fig. 1B, C) and 6 months old animals (Fig. 1D, E). In both age groups, cartilage area was smaller in Mc1re/e mice compared to WT which was statistically significant. Notably, we were unable to detect any signs of spontaneous OA-related cartilage surface erosions.

### Analysis of cartilage degradation during OA-pathogenesis

**Figure 2:**
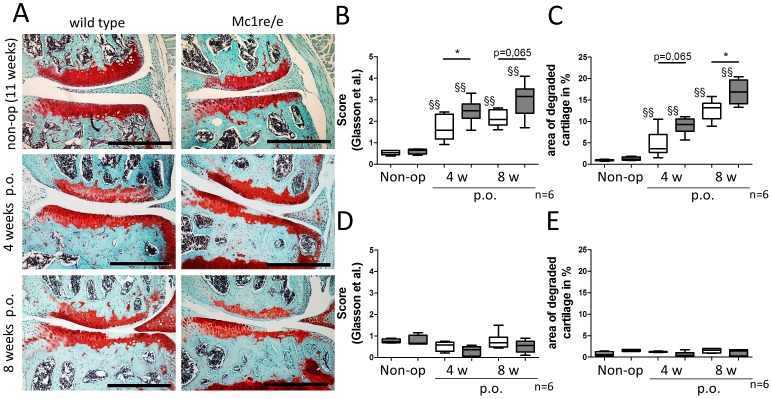
Assessment of cartilage degradation 4 and 8 weeks after osteoarthritis induction. A-E) Frontal sections of knee joints from WT and Mc1re/e mice of non-operated (non-op) 11 week old animals, and of animals 4 and 8 weeks post operation (p.o.) by DMM were stained with Safranin O/Fast green. A) Representative images of the medial area from sections of right knee joints from non-operated 11 weeks old animals and from mice 4 and 8 weeks after osteoarthritis induction (p.o.) show disease progression over the time. B–E) 5–6 sections in 60–80 µm intervals of right knees (DMM, B, C) and left knees (Sham, D, E) were scored histological according to Glasson et al. [21] (B, D) and morphometrically (C, E). B) We observed OA-progression over time in WT (p = 0.0043) and Mc1re/e (p = 0.0095) mice compared to non-operated controls. Mc1re/e mice had higher scores 4 weeks (p = 0.0411) and 8 weeks (p = 0.0649) after OA-induction. C) Percentages of degraded cartilage area also indicated an OA-progression over time in WT (p = 0.0043) and Mc1re/e (p = 0.0095) compared to non-operated controls. Mc1re/e mice had more degraded cartilage 4 weeks (p = 0.0649) and 8 weeks (p = 0.0411) after OA-induction compared to WT animals. D+E) Sham operated knee joints showed similar scores (D) and area of degraded cartilage (E) compared to non-operated controls. Mean scores of medial tibia and femur were included in statistical analysis. Data are presented as box plots reflecting the 25th and 75th percentile as boxes, the median as horizontal line and minimum and maximum values as whiskers. White bars indicate wild type and grey bars indicate mutant group. Bars = 500 µm, §§ p<0.01 4/8 weeks post-surgery vs. non-operated, * p<0.05 wild type vs. Mc1re/e.

Scoring and histomorphometric analysis of Safranin O stained articular cartilage revealed that OA was successfully induced in right knee joints of both Mc1re/e and WT mice (Fig. 2A). Both evaluation methods indicated significant increase in severity of cartilage degradation during OA progression in both groups. OA scoring of non-operated Mc1re/e and WT mice reveal that there is no spontaneous osteoarthritis induction in 11 weeks old (non-op; Fig. 2B–E) and 6 months old (data not shown) animals. Osteoarthritic related cartilage alterations as increasing loss of ECM macromolecules and cartilage surface erosions were more severe in Mc1re/e knee joints 4 and 8 weeks after OA induction (p.o.) for both evaluation methods (Fig. 2B, C) while sham-operated knees were unaffected (Fig. 2D, E).

### Distribution of MC1 in DMM- and non-operated knee joints of Mc1re/e and WT mice

**Figure 3:**
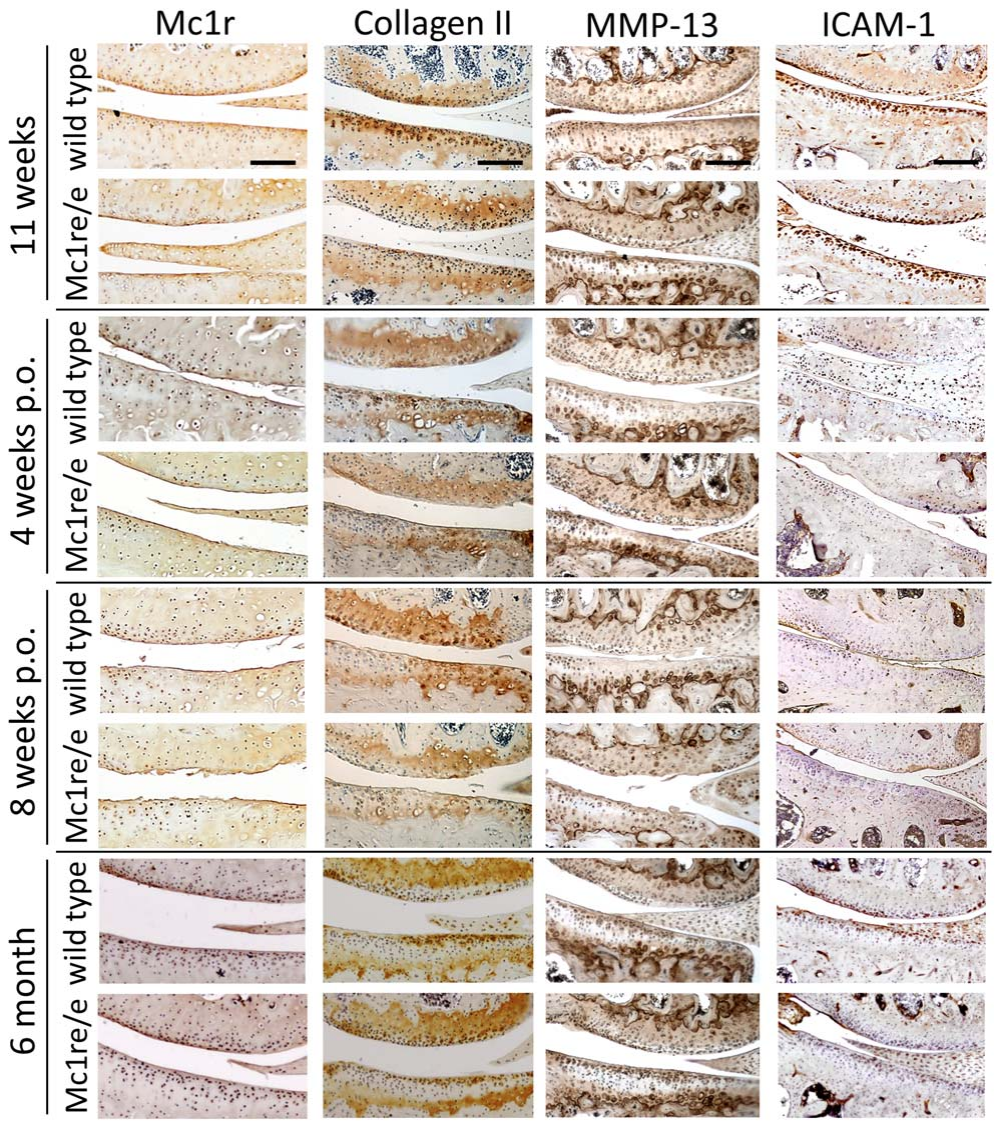
Localization of MC1, collagen II, MMP-13 and ICAM-1 in knee joints of Mc1re/e and wild type mice. A+C) Frontal sections of right knee joints from WT and Mc1re/e mice of non-operated (non-op) 11 weeks old and 6 months old animals, and of mice 4 and 8 weeks after osteoarthritis induction (post operation (p.o.)) were stained with antibodies against MC1, collagen II, MMP-13 and ICAM-1 as described in material and methods. Representative pictures of medial parts of the knee joints from each time point are shown. Bars = 200 µm.

Immunoreactivity for MC1 was tested on sections of knee joints from non-operated 11 week old and 6 month old Mc1re/e and WT animals and from mice 4 and 8 weeks after OA-induction with DMM. Representative images are shown in Figure 3.

Immunostaining demonstrates the presence of MC1 in knee joints of mice. Chondrocytes in menisci and the upper and the adjunct part of the middle zone of articular cartilage are immunopositive for MC1. In addition, chondrocytes of the growth plate stain positive for MC1, here mainly cells in the proliferating zone which are organized into columns are stained (Fig. S2). Mostly, hypertrophic chondrocytes and also subpopulations of bone cells in the subchondral bone area remain negative. There is no difference in staining intensity or profile between WT and Mc1re/e mice detectable after OA-induction. Also, in 11 weeks and 6 months old non-operated mice MC1 distribution appears not to be altered between mutant and WT group and during aging (Fig. 3).

### Collagen II, MMP-13, ICAM-1 and DIPEN in DMM- and non-operated knee joints of WT and Mc1re/e mice

**Figure 4:**
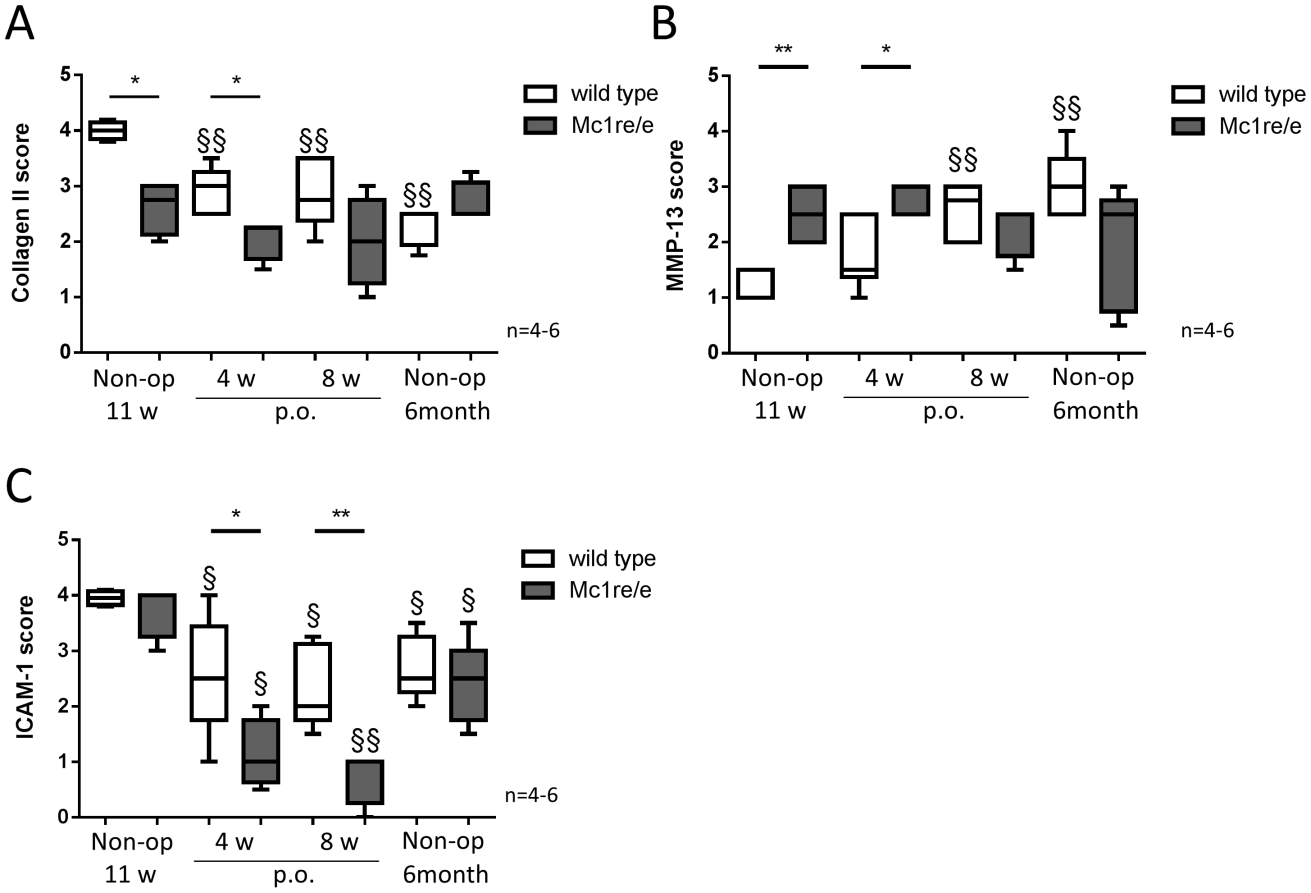
Quantitative scoring of collagen II, MMP-13 and ICAM-1 immunohistochemistry. Medial parts of frontal sections of right knee joints from WT and Mc1re/e mice of non-operated (non-op) 11 weeks old and 6 months old animals, and of mice 4 and 8 weeks after osteoarthritis induction (post operation (p.o.)) were scored after staining. 1-3 sections of right knees (DMM) were scored histological according to the scoring system illustrated in Tab. 1 for collagen II, in Tab. 2 for MMP-13 and in Tab. 3 for ICAM-1 staining. A) Compared to non-operated controls collagen II stained cartilage area of WT knee joints is decreased 4 weeks (p = 0.0079) and 8 weeks (p = 0.0043) after OA-induction as well as in non-operated 6 month old animals (p = 0.0159). Sections of Mc1re/e knee joints showed no difference over time. Collagen II stained cartilage area of non-operated 11 weeks old Mc1re/e mice (p = 0.0159) and Mc1re/e mice 4 weeks (p = 0.0159) after OA induction is decreased compared to WT mice. B) Compared to non-operated controls number of MMP-13 positive non-hypertrophic articular chondrocytes of WT knee joints is increased 8 weeks (p = 0.0022) after OA-induction as well as in non-operated 6 month old animals (p = 0.0076). Sections of Mc1re/e knee joints showed no difference over time. MMP-13 positive chondrocytes of non-operated 11 weeks old Mc1re/e mice (p = 0.0079) and Mc1re/e mice 4 weeks (p = 0.035) after OA induction are increased compared to WT mice. C) Compared to non-operated controls number of ICAM-1 positive articular chondrocytes of Mc1re/e and WT knee joints are decreased 4 weeks (Mc1re/e: p = 0.0159; WT: p = 0.0381) and 8 weeks (Mc1re/e: p = 0.0159; WT: p = 0.0159) after OA-induction as well as in non-operated 6 month old animals (Mc1re/e: p = 0.0317; WT: p = 0.0159). There is no difference between WT and Mc1re/e regarding the number of ICAM-1 positive chondrocytes in non-operated controls whereas Mc1re/e mice 4 (p = 0.0303) and 8 weeks (p = 0.0079) after OA-induction showed lower numbers of ICAM-1 positive cells. Mean scores of medial tibia and femur were included in statistical analysis. Data are presented as box plots reflecting the 25th and 75th percentile as boxes, the median as horizontal line and minimum and maximum values as whiskers. §§ p<0.01 4/8 weeks post-surgery vs. non-operated, § p<0.05 4/8 weeks post-surgery vs. non-operated, ** p<0.01 wild type vs. Mc1re/e, * p<0.05 wild type vs. Mc1re/e.

Immunoreactivity for collagen II, MMP-13, ICAM-1 and DIPEN was tested on sections of knee joints from non-operated 11 week old and 6 month old Mc1re/e and WT animals and from mice 4 and 8 weeks after OA-induction with DMM. Representative images of MC1, collagen II, MMP-13 and ICAM-1 were shown in figure 3. Additionally a 0–4 point scoring system was applied for all groups and antigens (Tab. 1, 2 and 3) and data are presented for collagen II (Fig. 4A,), MMP-13 (Fig. 4B) and ICAM-1 (Fig. 4C) staining. Images of DIPEN staining and quantitative scoring is shown in Fig. S3. Immunohistology with appropriate isotype control antibodies served as negative control and remained unstained (Fig. S1).

In sections of 6 month old non-operated WT animals and of WT mice 4 and 8 weeks after OA-induction, collagen II stained articular cartilage is significantly decreased compared to non-operated 11 week old control. In contrast, articular cartilage of Mc1re/e showed no loss of collagen II staining in sections of 6 month old non-operated WT animals and of WT mice 4 and 8 weeks after OA-induction compared to non-operated 11 week old controls. Between non-operated 11 weeks old Mc1re/e and WT mice, collagen II stained knee joints revealed differences in stained cartilage area with significantly less collagen II staining in Mc1re/e joints. Also, 4 weeks after OA-induction there are significantly more cartilage regions without collagen II staining in Mc1re/e mice compared to WT. At 8 weeks after DMM this is reflected by trend only. At 6 month of age, collagen II staining pattern did not differ between WT and Mc1re/e mice (Fig. 3 and Fig. 4A). Cohens kappa coefficients showed a substantial (MFC: 0.64) and moderate (MTP: 0.55) intra-observer agreement and a moderate (MFC: 0.40, MTP: 0.46) inter-observer agreement (Fig. S4).

Immunoreactivity for MMP-13 was significantly increased in MMP-13 positive non-hypertrophic articular chondrocytes 8 weeks after OA-induction and in sections of non-operated 6 months old animals compared to non-operated 11 weeks old mice. In contrast, articular chondrocytes of Mc1re/e did not show an increase of MMP-13 positive cells during OA-progression or ageing compared to 11 weeks old non-operated mice. We observed a significant higher number of MMP-13 positive non-hypertrophic chondrocytes in Mc1re/e mice compared to WT in non-operated 11 weeks old animals. This difference persisted 4 weeks after OA-inductions and was not observed 8 weeks after OA-induction and in non-operated 6 month old animals (Fig. 3 and Fig 4B). Cohens kappa coefficients showed a fair (MFC: 0.25) and moderate (MTP: 0.50) intra-observer agreement and a moderate (MFC: 0.40, MTP: 0.46) inter-observer agreement (Fig. S4).

Notably, staining for ICAM-1 revealed strong signals in chondrocytes of the articular cartilage and in chondrocytes mainly of the hypertrophic zone of the growth plate (Fig. S2). In WT and Mc1re/e animals, 6 month old non-operated animals and mice 4 and 8 weeks after OA-induction contain fewer ICAM-1 positive articular chondrocytes compared to non-operated 11 week old control. We detected no differences in ICAM-1 staining between Mc1re/e and WT mice in non-operated 11 weeks old and 6 month old animals. 4 and 8 weeks after OA-induction, Mc1re/e mice showed significantly less ICAM-1 positive articular chondrocytes in the medial part of the joints compared to WT (Fig. 3 and Fig. 4C). Cohens kappa coefficients showed a substantial (MFC/MTP: 0.74) intra-observer agreement and a fair (MFC: 0.37, MTP: 0.32) inter-observer agreement (Fig. S4).

Staining for MMP-generated aggrecan neoepitope DIPEN revealed highest immunoreactivity in articular cartilage chondrocytes of 11 weeks old WT and Mc1re/e mice. 4 weeks after OA-induction, number of DIPEN positive chondrocytes was lower compared to 11 weeks old mice of both groups. Numbers of DIPEN positive chondrocytes did not differ between WT and Mc1re/e in age-matched mice and 4 and 8 weeks after OA-induction (Fig. S3). Cohens kappa coefficients showed a substantial (MFC: 0.61, MTP: 0.71) intra-observer agreement and a fair (MFC: 0.26, MTP: 0.36) inter-observer agreement (Fig. S4).

### Analysis of bone architecture at the day of surgery (day 0)

**Figure 5:**
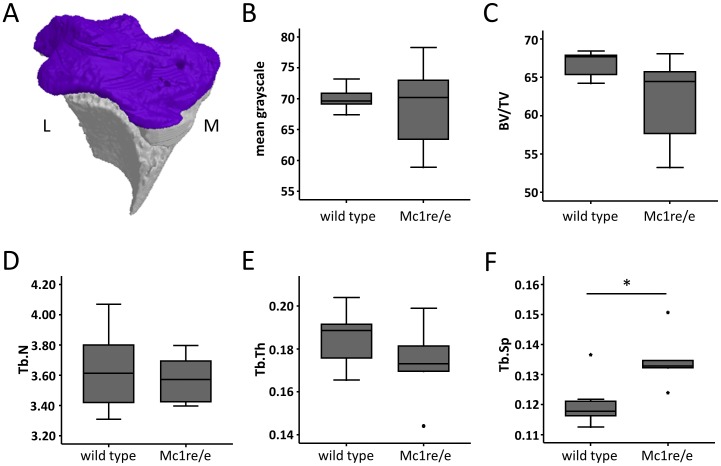
Comparison of bone architecture between Mc1re/e and WT mice at the day of DMM surgery (day 0) using µCT. A–F) µCT 3D analysis of bone parameters was performed within a defined VOI including the tibia epiphysis of both knees respectively. A) Frontal view on 3D model of the proximal tibia of the right leg showing the VOI marked in purple (L: lateral, M: medial). B) µCT analysis of mean grayscale within the VOI as an indicator for bone density indicated no difference between WT and Mc1re/e mice. C) 3D calculation of BV/TV within the VOI as an indicator of bone mass also revealed no difference between both groups. In Trabecular number (Tb.N, D) and trabecular thickness (Tb.Th, E) no difference was observed whereas trabecular separation (Tb.Sp, F) was increased in Mc1re/e compared to controls (p = 0.028). Values of left and right knees were combined. Data are presented as boxplots reflecting the 25th and 75th percentile as boxes, the median as horizontal line and minimum and maximum values as whiskers, stars indicating extreme outliers. * p<0.05 wild type vs. Mc1re/e.

Bone morphology of the epiphysis of the right tibia was recorded at the day of surgery (day 0) by µCT analysis within a VOI excluding the growth plate (Fig. 5A). The mean grayscale was calculated as an indicator for bone density (Fig. 5). Bone volume (BV) was calculated relative to total volume (TV) to normalize on knee size and was used as an indicator for bone mass (Fig. 5C). Bone density and BV/TV were similar between WT and Mc1re/e mice at the day of surgery indicating no differences in epiphyseal bone density and mass prior to OA induction. Trabecular number (Tb.N, Fig. 5D) and trabecular thickness (Tb.Th, Fig. 5E) did not differ between the groups at day 0 whereas mutants showed an increased trabecular separation (Tb.Sp, Fig. 5F).

### Development of osteophytes in the joints of Mc1re/e mice after OA-induction

**Figure 6:**
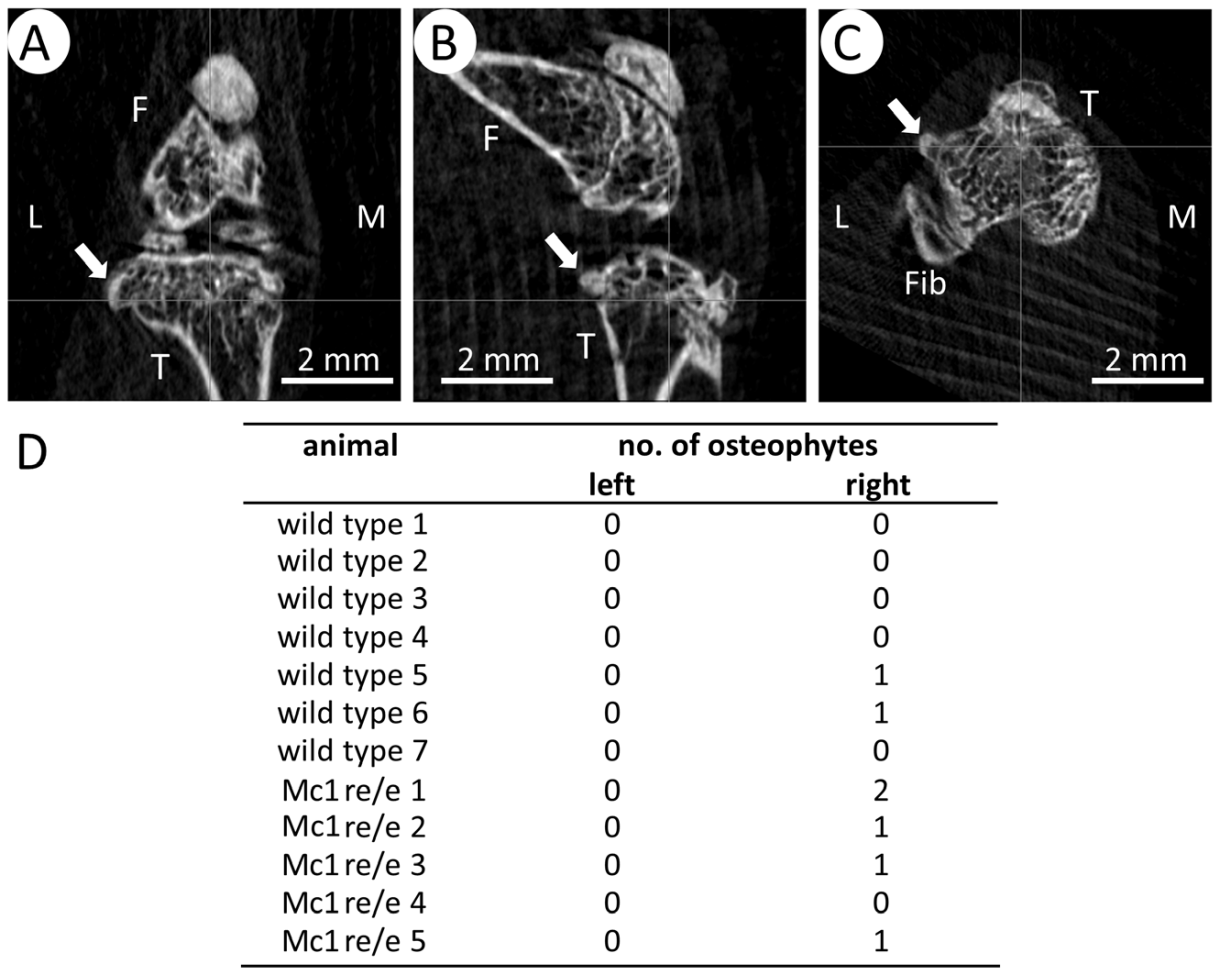
Radiographic evaluation of osteophytes at 4 and 8 weeks past OA induction. A–C) Knees were oriented as described in material and methods section and were analyzed for osteophytes (white arrow) in transaxial (A), sagittal (B) and coronal (C) plane. F: femur, T: tibia, Fib: fibula, L: lateral and M: medial. D) At 4 and 8 weeks the number and size of osteophytes was calculated for the right and the left knee of each animal, respectively. 4 of 5 Mc1re/e mice developed osteophytes whereas only 2 from 7 WT mice developed one osteophyte after OA-induction.

Osteophytes were evident 4 weeks after OA-induction in 2 of 7 animals of the WT- and in 4 of 5 animals of the Mc1re/e group (Fig. 6A–C). Only the right knee (DMM) was affected, sham-operated knees did not develop osteophytes. Osteophytes were increased in size at 8 weeks, but no additional novel osteophytes became apparent 8 weeks after OA-induction (Fig. 6D).

### Increased bone density and bone volume in knees of Mc1re/e mice after OA-induction

**Figure 7:**
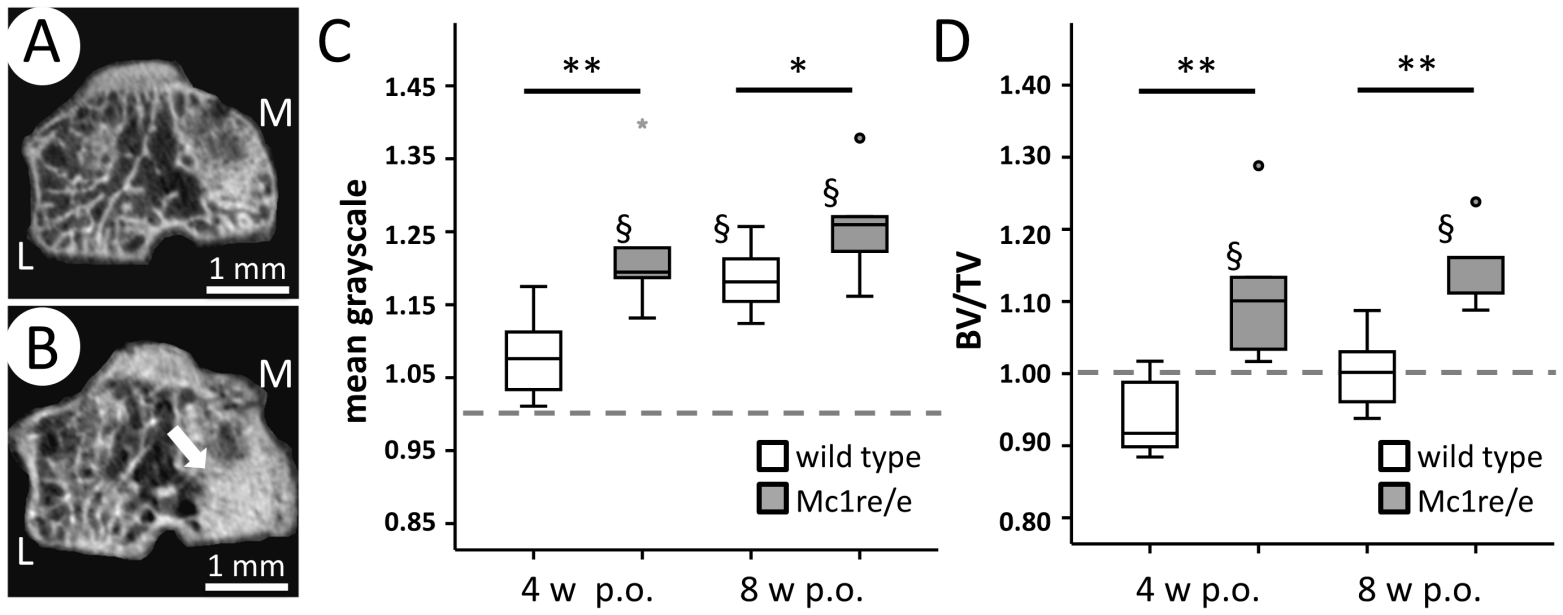
Alteration of bone density and bone volume at 4 and 8 weeks past OA induction. A–B) Reconstructed coronal image of Mc1re/e knee joints at the day of OA-surgery (day 0) (A) and 8 weeks after OA-surgery (B). White arrow marks an area of increased mineral deposition (L: lateral, M: medial). C) µCT analysis of mean grayscale within the VOI of the right knees as an indicator for bone density revealed an increase in bone densitiy 4 (p = 0.043) and 8 weeks (p = 0.043) after OA-indution in Mc1re/e mice and 8 weeks (p = 0.018) after OA-induction in WT animals. Mc1re/e mice showed a higher bone density 4 (p = 0.007) and 8 weeks (p = 0.042) after OA-induction compared to WT mice. D) In Mc1re/e mice but not in WT animals, BV/TV within the VOI of the right knee as an indicator of bone mass was increased at both time points after OA-induction compared to day 0 (p = 0.043). There was a higher bone mass in the epiphysis of Mc1re/e mice 4 (p = 0.007) and 8 weeks (p = 0.004) after OA-induction compared to WT animals. Data were normalized to the values at day 0 (day of surgery, (set as 1, grey dotted line). Data are presented as boxplots reflecting the 25th and 75th percentile as boxes, the median as horizontal line and minimum and maximum values as whiskers with dots indicating outliers and stars indicating extreme outliers. § p<0.05 4/8 weeks post-surgery vs. day 0, * p<0.05 and ** p<0.01 wild type vs. mutant.

DMM-knees developed areas of increased mineral deposition during OA progression according to reconstructed images (Fig. 7A, B). At 4 weeks the mean grayscale as an indicator for bone density was significantly increased in the Mc1re/e group compared to day 0. Eight weeks after OA-induction WT animals revealed a significantly elevated bone density. At both time points, Mc1re/e mice had higher bone density in the epiphysis (Fig. 7C). BV/TV as an indicator of bone mass increased in the knee joints of the Mc1re/e group only compared to day 0 and was higher compared to the WT group 4 weeks and 8 weeks after OA-induction (Fig. 7D).

### Alterations in bone architecture are significant more pronounced in Mc1re/e mice affecting also the contra-lateral knee joints

**Figure 8:**
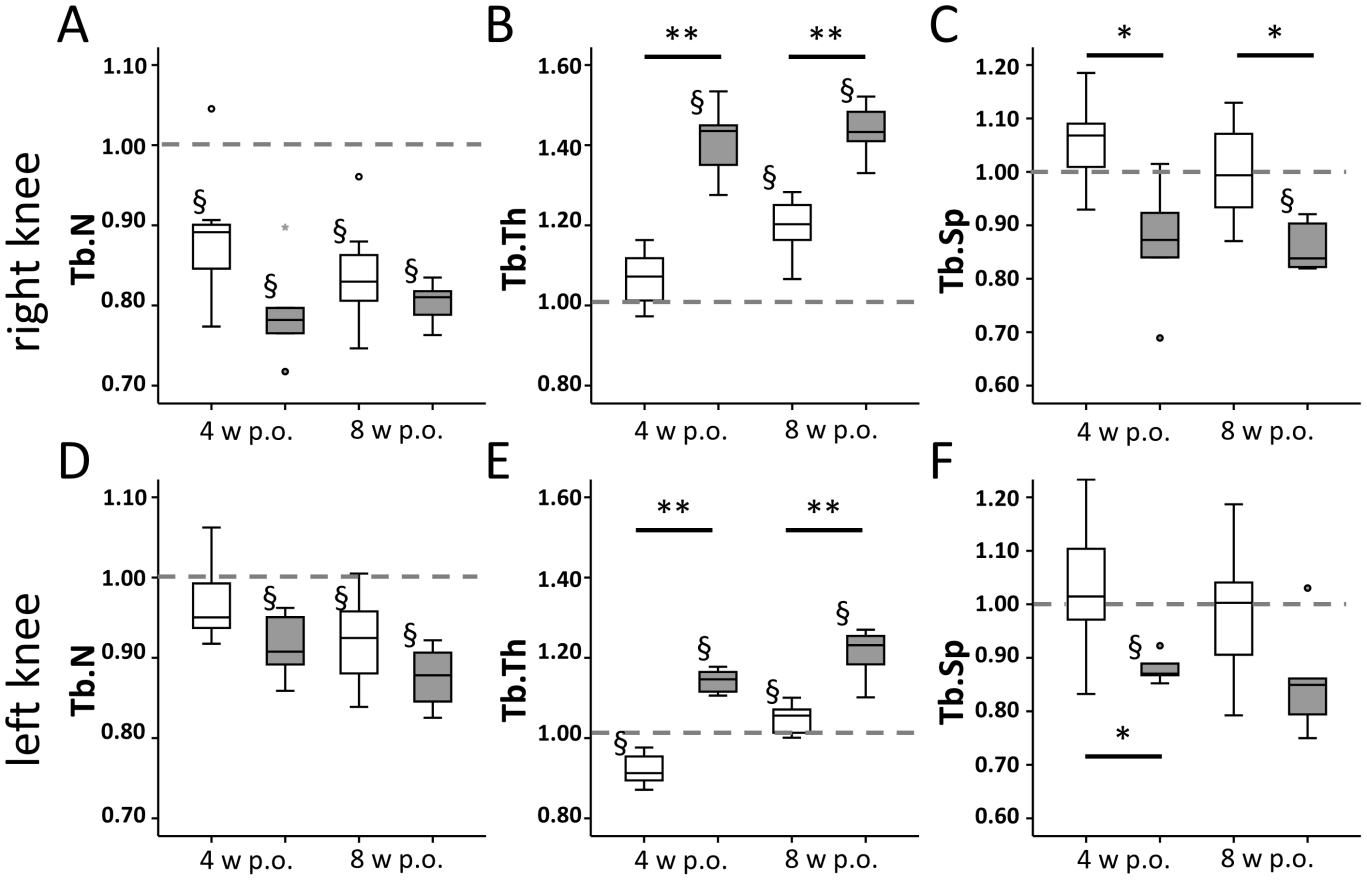
Alteration of bone architecture after OA induction. µCT 3D-analysis of trabecular parameters in the right knee joint (DMM, A–C) and the left knee joint (Sham, D–F) depicted as trabecular number (Tb.N, A,D), trabecular thickness (Tb.Th, B,E) and trabecular separation (Tb.Sp, C,F). A) Tb.N declined in WT (p = 0.018) and Mc1re/e (p = 0.043) at both time points after OA-induction compared to day 0 with no differences observed between both groups. B) Compared to day 0 animals Tb.Th was increased in Mc1re/e mice at both time points (p = 0.043) after OA-induction whereas WT mice showed an increase only 8 weeks after operation (p = 0.018). Mc1re/e mice had thicker trabeculae at both time points compared to WT animals (p = 0.004) C) Th.Sp was decreased in Mc1re/e animals reaching significance 8 weeks after OA-induction compared to day 0 mice (p = 0.043). At both time points, Mc1re/e animals developed less Th.Sp compared to WT group (p = 0.019). Mild alterations in trabecular structure also occurred in sham operated contra-lateral knees in both groups but to a significantly lesser extend compared to destabilized knees. Data were normalized to the values at day 0 (day of surgery, set as 1, grey dotted line). Data are presented as box plots reflecting the 25th and 75th percentile as boxes, the median as horizontal line and minimum and maximum values as whiskers with dots indicating outliers and stars indicating extreme outliers. White bars indicate wild type and grey bars indicate Mc1re/e group. § p<0.05 4/8 weeks post-surgery vs. day 0, * p<0.05 and ** p<0.01 wild type vs. mutant.

In destabilized DMM knee joints, trabecular number (Tb.N) declined significantly in WT and Mc1re/e at 4 and 8 weeks compared to day 0 with no differences observed between both groups (Fig. 8A).

Trabecular thickness (Tb.Th) was significantly increased in DMM-knees of the Mc1re/e group at 4 and 8 weeks compared to day 0. In the WT group an increase in Tb.Th was evident only at 8 weeks with trabeculae being significantly thinner compared to the Mc1re/e group at both time points (Fig. 8B).

In line with an increase of Tb.Th was a decrease in Tb.Sp in DMM knee joints of the Mc1re/e group reaching significance at 8 weeks compared to day 0. This decrease was not observed in the WT group at these time points. At both time points, Mc1re/e animals showed less Tb.Sp versus the WT group (Fig. 8C).

Interestingly, mild alterations in trabecular structure also occurred in sham operated contra-lateral knees in both groups within the set time span (Fig. 8D–E) but to a significantly lesser extend compared to destabilized knees. However, no significant changes in BV/TV were detected in contra-lateral knees of animals of both groups (data not shown).

## Discussion

Hallmark features of OA are structural changes including cartilage destruction as well as alterations in synovial membrane and subchondral bone. Synovial tissue and subchondral bone are considered to play decisive roles in OA-pathology, but up to now cartilage is the main target for therapeutic approaches because of its paramount importance in joint articulation. However, current therapies are palliative and there are no disease-modifying drugs available for effective clinical use. One major reason for the lack of curative therapies is that OA is mostly diagnosed in late or end stage of the disease where joint replacement by endoprotheses remains the only treatment option [26]. Thus, it is of great importance to understand basic mechanisms of OA-pathology in an early stage of the disease in order to develop effective regenerative therapies. For that approach we have chosen surgical OA induction with DMM in mice which allows to detect alteration in cartilage and subchondral bone already early after induction of OA and resembles quite closely slowly progressive human OA [19].

There is compelling evidence that the osteoarticular system is a direct target organ and source of POMC peptides [12]. Our study provides novel evidence for a role of the POMC system in the osteoarticular system and in OA-pathology. Mice which lack a functional MC1 develop a cartilage phenotype which is reflected in a smaller cartilage area, lower immunoreactivity for collagen II and a higher number of MMP-13 positive chondrocytes. After OA-induction, they develop more severe cartilage erosions and tissue loss compared to WT. Of note, immunoreactivity for ICAM-1 decreases during OA progression which is significantly more pronounced in articular chondrocytes of Mc1re/e mice as in WT. This effect supports our observation that MC1 signaling helps to stabilize cartilage matrix integrity. Immunostaining for MC1 revealed no difference between WT and MC1re/e mice. Reduced collagen II and increased MMP-13 immunostaining in non-operated Mc1re/e knees indicates a cartilage phenotype in the mutant mice independent of OA pathology suggesting a premature appearance of age – related structural ECM alterations in the absence of a functional MC1.

With respect to epiphyseal bone architecture, we did not detect obvious differences between Mc1re/e and WT mice regarding bone density and mass at the day of DMM surgery. However, trabecular separation was increased in Mc1re/e mice indicating a slight, but distinct effect on bone marrow thickness. We cannot exclude compensatory effects of other MCR subtypes in bone tissue, i.e. MC2 and MC4, for which at least mRNA transcripts are found in osteoblasts [17], [27]. In addition to matrix producing osteoblasts, bone contains also matrix degrading cells, the osteoclasts which express MC2, MC3, MC4 and MC5 transcripts [17]. Presumably, it needs challenging by inflicting traumata in order to reveal physiological relevance of an individual MCR subtype. With respect to cartilage morphology, histomorphometry demonstrates that the area in knee joints covered by articular cartilage surface was smaller in non-operated Mc1re/e mice compared to WT. This observation indicates, together with less collagen II stained area and a higher number of MMP-13 positive chondrocytes in sections of knee joints of 11 weeks old mutant mice, an OA – independent cartilage phenotype, however without signs of OA typical surface erosions. Notably, immunoreactivity for cellular MMP-generated aggrecan neoepitope DIPEN does not differ between WT and Mc1re/e in articular chondrocytes at any time point. Besides DIPEN, various other aggrecan neoepitopes generated by MMPs and aggrecanases are known which indicate aggrecan degradation. Our data suggest that presumably MMP-13 might not be the major proteinase causative for reduction of area of safranin O stained cartilage in Mc1re/e mice. Again, there might be some compensation as mRNA transcripts of other MCR subtypes, i.e. MC2 and MC5 were detected in articular cartilage from newborn mice (data not shown). In contrast to bone, chondrocytes are the only cell type in cartilage and for them MC1 signaling might be more important than signaling through other MCR subtypes. Recently, we demonstrated that α-MSH, the high affinity ligand of MC1, modulated metabolism of articular chondrocytes by altering gene expression and protein secretion of several collagens, MMPs and cytokines [15].

In this study we provide evidence that the POMC system can be chondro-osseo protective and ameliorates OA pathogenesis. In vivo effects of melanocortin peptides which affect the osteoarticular system are mainly anti-inflammatory and affect bone turnover and -volume. α-MSH, the high-affinity ligand of MC1 besides ACTH [12], is reported to reduce collagen-induced arthritis in mice [28] and to lead to reduced arthritis scores as well as reduced articular erosions in rat adjuvant arthritis [29]. Interestingly, α-MSH also reduces tibial perimeter and length. In primary cultures of osteoblasts and chondrocytes, α-MSH dose dependently stimulated cell proliferation while in bone marrow cultures, α-MSH stimulated osteoclastogenesis. Systemic administration of α-MSH to mice decreased trabecular bone volume in the proximal tibiae and reduced trabecular number. From this it can be concluded that α-MSH acts directly on bone, increasing bone turnover, and, when administered systemically, decreasing bone volume [30]. These observations are nicely in line with our data showing increased bone density and bone mass in MC1-signaling deficient mice after OA-induction. With respect to cartilage matrix formation, α-MSH and ACTH stimulate matrix production by increasing collagen II and aggrecan expression in committed murine and rat chondrocytes [14], [15]. This anabolic effect of melanocortins would help to explain our observation of a articular cartilage phenotype in native Mc1re/e mice and of more severe cartilage matrix degradation and loss in OA.

Together with our observation that functional MC1 signaling delays cartilage degradation and loss during experimental OA pathogenesis, we also observed alterations in subchondral bone micro-architecture and osteophyte number in MC1 signaling-deficient mice after induction of OA. Mutant mice develop clearly more and larger osteophytes as WT. Osteophyte formation is besides joint space narrowing, subchondral sclerosis and subchondral cyst formation one of the main radiographic features of OA and an important criterion for this disease. Osteophytes have a significant clinical impact and can be a source of pain and loss of function [31]. In addition to increased osteophyte formation, lack of MC1 signaling leads to increased subchondral bone mass and bone density after OA-induction. In early OA, a marked thinning, increase in porosity [23] and hypomineralization of subchondral bone is observed due to an abnormal high turnover [32] while in late OA-stages subchondral bone thickening occurs followed by sclerosis [33]. In line with a significant increase in trabecular thickness and decrease of trabecular separation, our observations indicate a more severe and clearly faster progression of subchondral OA-related sclerosis in Mc1re/e mice. Notably, we observed similar effects, however less pronounced in sham-operated knees at 4 and 8 weeks after DMM with increased severity in Mc1re/e mice. We were unable to detect OA related cartilage matrix alterations in sham-knees in both groups during that time points indicating that during early OA alterations in subchondral bone morphology precede OA related phenotypical changes in cartilage matrix.

We did not detect obvious differences in MC1 immunostaining between WT and Mc1re/e mice or during OA progression indicating that MC1 synthesis per se is not affected by the disease. Contrary to human OA-cartilage [15], mostly chondrocytes of the upper and partly the middle layer were MC1 positive while chondrocytes close to the tide mark remained unstained. These differences in MC1 distribution might be due to the profound thinner articular cartilage in mice which consists of only a few cell layers. Notably, in line with Zhong et al., we also found that chondrocytes mainly in the proliferative zone of the growth plate stained positive for the MC1 whereas chondrocytes of the hypertrophic zone lack not only MC1 expression but mRNA for all other MCR subtypes [17]. Our unpublished observations indicated an induction of ICAM-1 gene and protein expression in human articular OA-chondrocytes in vitro when stimulated with α-MSH which prompted us to analyze ICAM-1 protein expression in mouse joints with immunohistochemistry. Notably, we found strong ICAM-1 immunoreactivity in articular chondrocytes of non-operated 11 week old Mc1re/e and WT mice which was decreased in 6 month old animals. Moreover, during pathogenesis of OA, in cartilage of Mc1re/e mice even less ICAM-1 positive chondrocytes were detected compared to WT. ICAM-1 is constitutively expressed on articular chondrocytes [34] and is a cell surface receptor for hyaluronan (HA) [35], [36]. Hyaluronan and collagen II are two major components of cartilage ECM which, among other roles, serve as structural components for cell adhesion. One can speculate that imbalance of these ECM molecules feeds back on the expression of associated adhesion molecules like ICAM-1. Loss of ICAM-1 expression after OA-induction could add to the aggravated degradation of cartilage in Mc1re/e mice when challenged with DMM. The study of Yasuda et al. indicate that the interaction of ICAM-1 and HA suppresses cartilage destruction induced via collagen II peptides and MMP-13 production [37] suggesting that increased loss of ICAM-1 in this context might facilitate OA pathology. Moreover, Lisignoli et al., demonstrated an anti-apoptotic effect of HA bound to its receptors CD44 and ICAM-1 [38] not only after OA induction but already in unchallenged mice.

### Conclusions

Inflammatory-like processes originated from OA increase bone and cartilage turnover and eventually lead to degradation of these tissues. The osteoarticular system presumably responds to this with an evolutionary conserved stress response in analogy to the classical HPA axis, that is increased synthesis of POMC-derived peptides. The release of such POMC peptides will subsequently not only modulate the activity of immune cells but also of resident cells as osteoclasts, osteoblasts, chondrocytes, synoviocytes and their progenitor cells which carry the appropriate MC-receptors. Inactivation of the MC1, which is present on chondrocytes and cells of epiphyseal bone will thus consequently have an effect on physiology and pathophysiology of diathrodial joint tissues. Our study demonstrates that signal-deficient Mc1re/e mice have a cartilage phenotype prior to OA induction which increases in severity during OA-pathogenesis already in an early stage. In addition, we suggest that absence of MC1-signaling accelerates age-related structural cartilage ECM alterations as demonstrated by loss of collagen II and increased number of MMP-13 positive chondrocytes. Notably, our data from sham – operated joints suggest that OA-pathogenesis related alterations in epiphyseal bone architecture precede alterations in articular cartilage which is more pronounced in Mc1re/e joints. Understanding the underlying molecular mechanisms of the functional role of the POMC system in joints will eventually help to tailor efficient future therapies against degenerative joint diseases as OA.

## Supporting Information

Figure S1 **Isotype control staining for MC1, collagen II, MMP-13, DIPEN and ICAM-1 immunohistochemistry.** Frontal sections of right knee joints from WT and Mc1re/e mice of non-operated 11 weeks old (Collagen II and DIPEN) and 6 months old (MC1, MMP-13 and ICAM-1) animals were stained with antibodies against MC1, collagen II, MMP-13, DIPEN and ICAM-1and appreciate isotype control antibodies. Representative pictures of medial parts of the knee joints from WT and Mc1re/e stained with specific antibodies and isotype control antibodies are shown. Staining with isotype control antibodies revealed no staining. Black bars = 200 µm.
(TIF)

Figure S2 **Localization of MC1, collagen II, MMP-13 and ICAM-1 in articular cartilage and tibial growth plate of Mc1re/e and WT mice.** Frontal sections of right knee joints from WT and Mc1re/e mice of non-operated 11 weeks old and 6 months old animals and of mice 4 and 8 weeks after OA-induction were stained with antibodies against MC1, collagen II, MMP-13 and ICAM-1 as described in material and methods. Representative pictures of a 520× magnification of medial, tibial articular cartilage and tibial growth plate of the knee joints from 11 weeks old (Collagen II, Col II and MMP-13) and 6 months old (MC1 and ICAM-1) mice are shown. Black bars = 200 µm, white bars = 25 µm.
(TIF)

Figure S3 **Localization of aggrecan neoepitope DIPEN in knee joints of Mc1re/e and wild type mice.** Frontal sections of right knee joints from WT and Mc1re/e mice of non-operated (non-op) 11 weeks old and 6 months old animals, and of mice 4 and 8 weeks after osteoarthritis induction (post operation (p.o.)) were stained with antibodies against DIPEN as described in material and methods. A) Representative pictures of medial parts of the knee joints from each time point are shown. B) Number of DIPEN positive chondrocytes was scored according to Tab. 3. Compared to 11 weeks old non-op mice number of DIPEN positive decreased 4 weeks after OA-induction in both groups (WT: p = 0,0186 and Mc1re/e: p = 0,0647). Mean scores of medial tibia and femur were included in statistical analysis. Data are presented as box plots reflecting the 25th and 75th percentile as boxes, the median as horizontal line and minimum and maximum values as whiskers. Bars = 200 µm; § p<0.05 4/8 weeks post-surgery vs. non-operated; (§) p<0.0647 4/8 weeks post-surgery vs. non-operated.
(TIF)

Figure S4 **Intra- and inter-observer agreement of histological scoring systems.** Histological scoring systems for collagen II (A–B), MMP-13 (C-D), ICAM-1 (E–F) and DIPEN (G–H) staining were evaluated. Therefore 7 independent observer scored 10 frontal sections of each staining to obtain inter-observer agreement and 4 observer scored twice within one week to get intra-observer variability. Summed scores of medial femoral condyle (MFC) and medial tibial plateau (MTP) were shown for collagen II (A), MMP-13 (C), ICAM-1 (E) and DIPEN (G) scoring. To determine intra-observer agreement, cohens kappa coefficient between scores of measurement 1 and scores of measurement 2 of each observer (MV, RB, SG and TN) was calculated. For inter-observer agreement, cohens kappa coefficient between each observer of measurement 1 was determined. Mean of cohens kappa coefficients of collagen II (B), MMP-13 (B), ICAM-1 (F) and DIPEN (H) scorings were shown.
(TIF)
