# Supplementary figures and images for: Melanocortin 1 Receptor-Signaling Deficiency Results in an Articular Cartilage Phenotype and Accelerates Pathogenesis of Surgically Induced Murine Osteoarthritis
Source: PLoS One. 2014 Sep 5;9(9):e105858. doi: 10.1371/journal.pone.0105858 (PMC4156302; doi:10.1371/journal.pone.0105858)

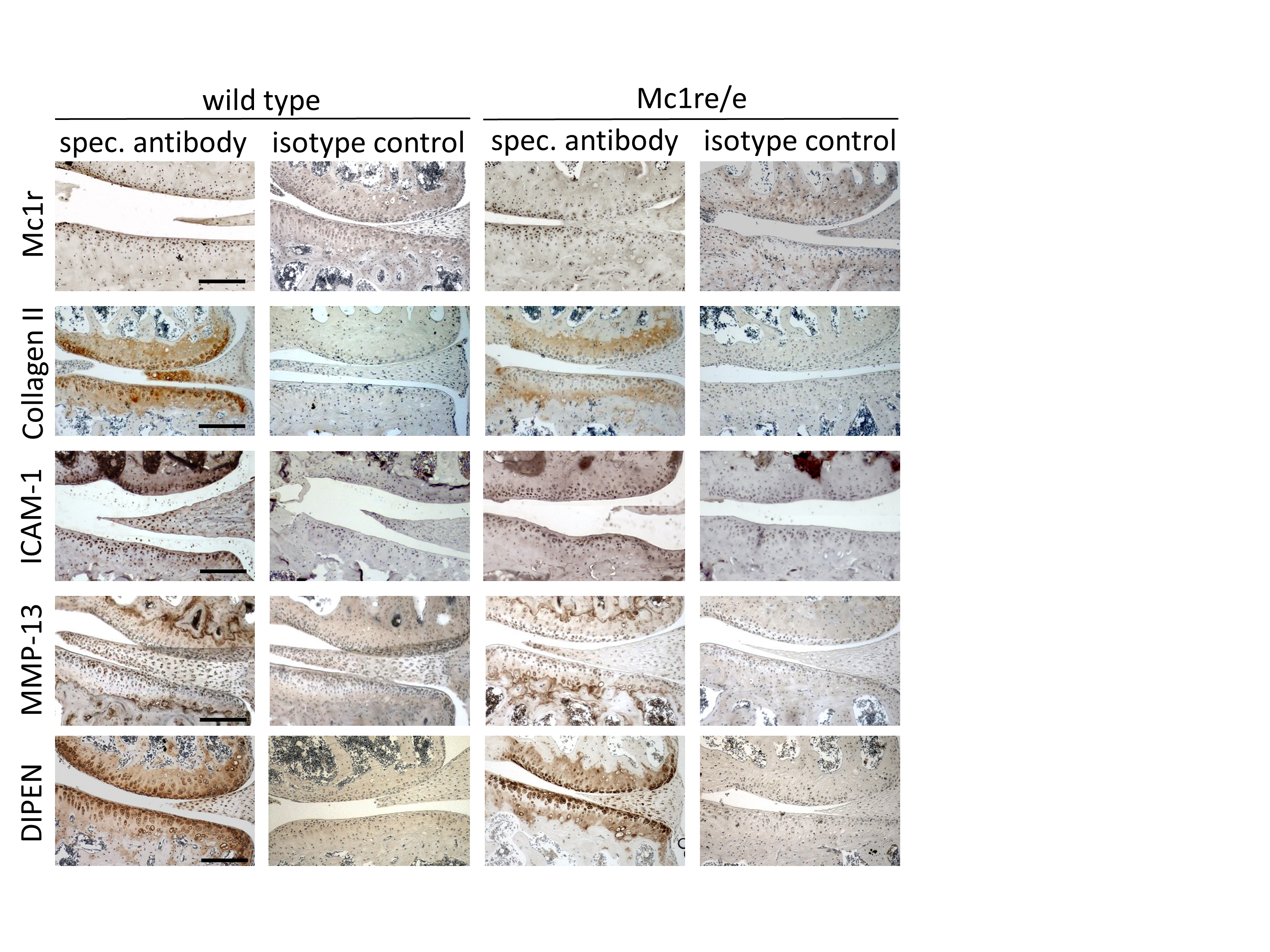

Supplement: Figure S1 — Isotype control staining for MC1, collagen II, MMP-13, DIPEN and ICAM-1 immunohistochemistry. Frontal sections of right knee joints from WT and Mc1re/e mice of non-operated 11 weeks old (Collagen II and DIPEN) and 6 months old (MC1, MMP-13 and ICAM-1) animals were stained with antibodies against MC1, collagen II, MMP-13, DIPEN and ICAM-1and appreciate isotype control antibodies. Representative pictures of medial parts of the knee joints from WT and Mc1re/e stained with specific antibodies and isotype control antibodies are shown. Staining with isotype control antibodies revealed no staining. Black bars = 200 µm. (TIF) [file pone.0105858.s001.tif]

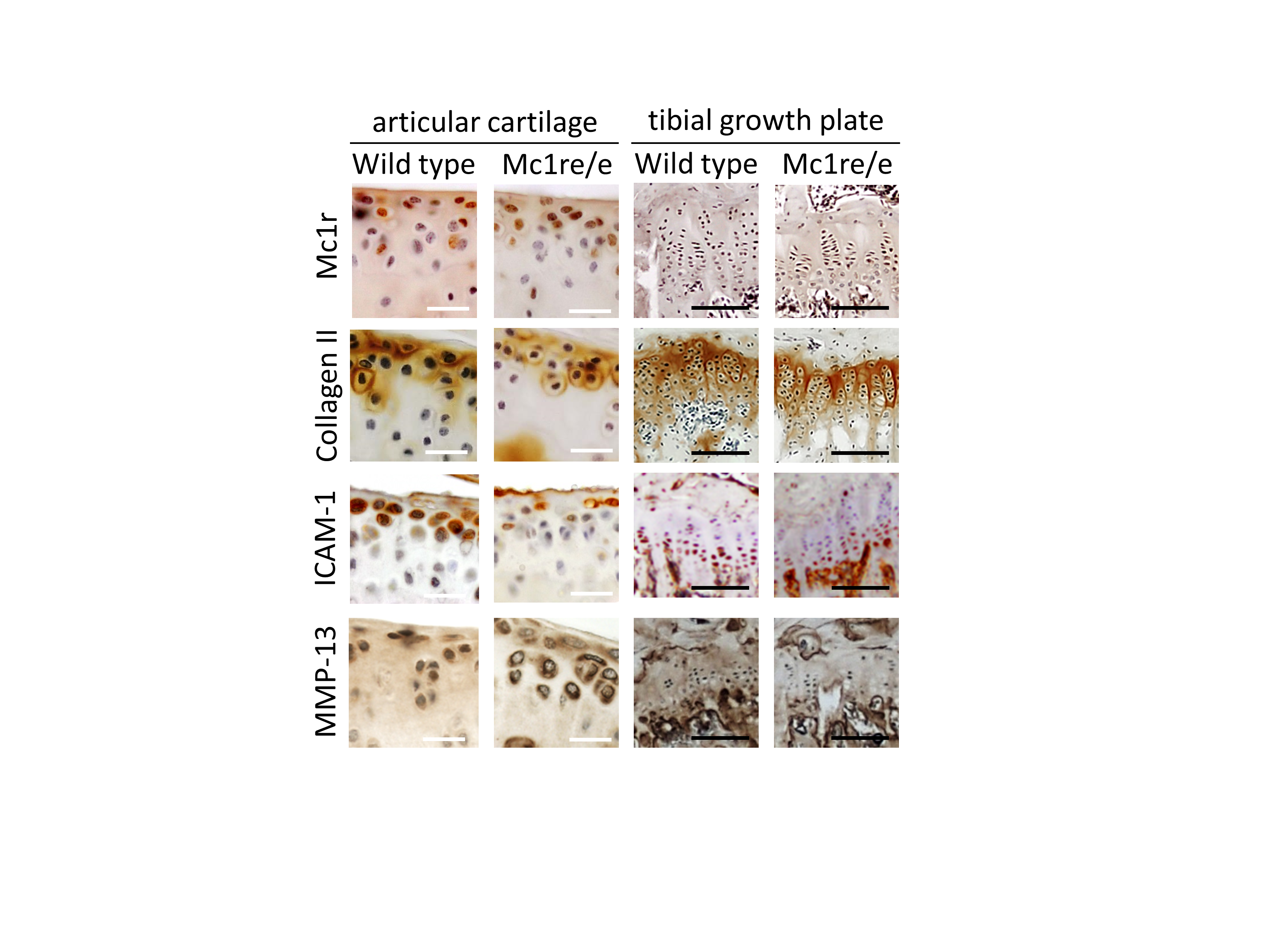

Supplement: Figure S2 — Localization of MC1, collagen II, MMP-13 and ICAM-1 in articular cartilage and tibial growth plate of Mc1re/e and WT mice. Frontal sections of right knee joints from WT and Mc1re/e mice of non-operated 11 weeks old and 6 months old animals and of mice 4 and 8 weeks after OA-induction were stained with antibodies against MC1, collagen II, MMP-13 and ICAM-1 as described in material and methods. Representative pictures of a 520× magnification of medial, tibial articular cartilage and tibial growth plate of the knee joints from 11 weeks old (Collagen II, Col II and MMP-13) and 6 months old (MC1 and ICAM-1) mice are shown. Black bars = 200 µm, white bars = 25 µm. (TIF) [file pone.0105858.s002.tif]

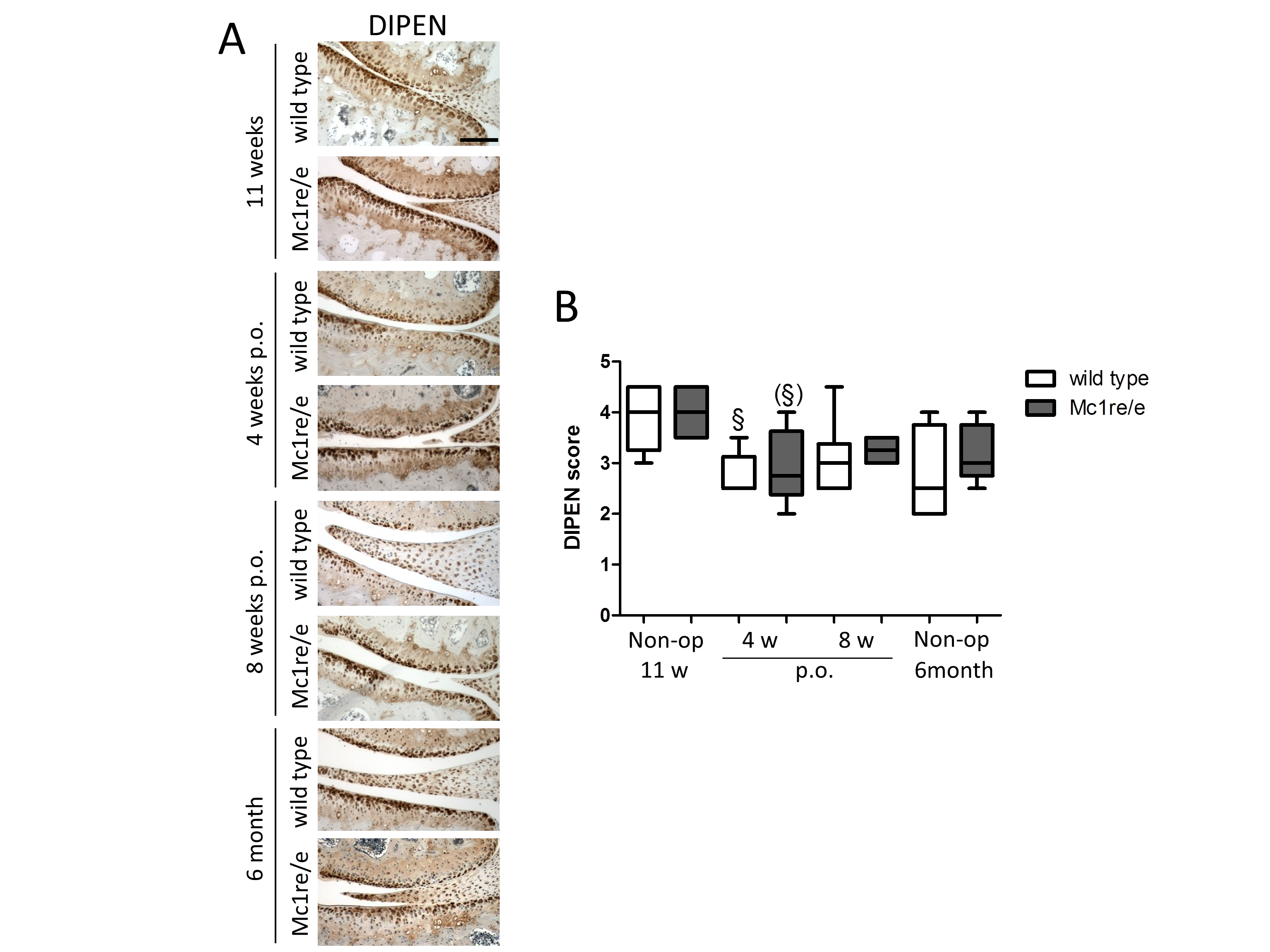

Supplement: Figure S3 — Localization of aggrecan neoepitope DIPEN in knee joints of Mc1re/e and wild type mice. Frontal sections of right knee joints from WT and Mc1re/e mice of non-operated (non-op) 11 weeks old and 6 months old animals, and of mice 4 and 8 weeks after osteoarthritis induction (post operation (p.o.)) were stained with antibodies against DIPEN as described in material and methods. A) Representative pictures of medial parts of the knee joints from each time point are shown. B) Number of DIPEN positive chondrocytes was scored according to Tab. 3. Compared to 11 weeks old non-op mice number of DIPEN positive decreased 4 weeks after OA-induction in both groups (WT: p = 0,0186 and Mc1re/e: p = 0,0647). Mean scores of medial tibia and femur were included in statistical analysis. Data are presented as box plots reflecting the 25th and 75th percentile as boxes, the median as horizontal line and minimum and maximum values as whiskers. Bars = 200 µm; § p<0.05 4/8 weeks post-surgery vs. non-operated; (§) p<0.0647 4/8 weeks post-surgery vs. non-operated. (TIF) [file pone.0105858.s003.tif]

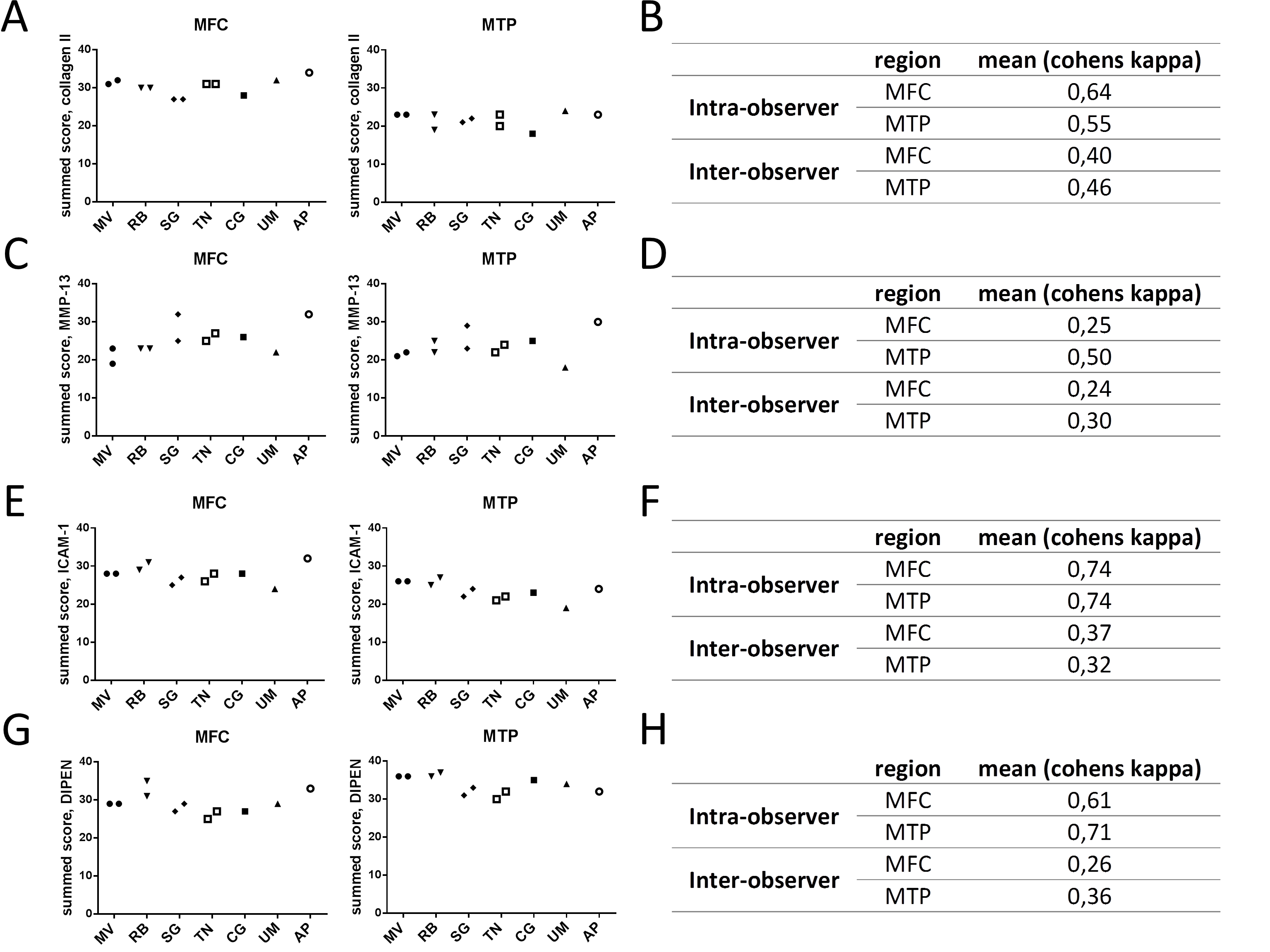

Supplement: Figure S4 — Intra- and inter-observer agreement of histological scoring systems. Histological scoring systems for collagen II (A–B), MMP-13 (C-D), ICAM-1 (E–F) and DIPEN (G–H) staining were evaluated. Therefore 7 independent observer scored 10 frontal sections of each staining to obtain inter-observer agreement and 4 observer scored twice within one week to get intra-observer variability. Summed scores of medial femoral condyle (MFC) and medial tibial plateau (MTP) were shown for collagen II (A), MMP-13 (C), ICAM-1 (E) and DIPEN (G) scoring. To determine intra-observer agreement, cohens kappa coefficient between scores of measurement 1 and scores of measurement 2 of each observer (MV, RB, SG and TN) was calculated. For inter-observer agreement, cohens kappa coefficient between each observer of measurement 1 was determined. Mean of cohens kappa coefficients of collagen II (B), MMP-13 (B), ICAM-1 (F) and DIPEN (H) scorings were shown. (TIF) [file pone.0105858.s004.tif]
